# Supramolecular Semiconductivity through Emerging Ionic Gates in Ion–Nanoparticle Superlattices

**DOI:** 10.1021/acsnano.2c07558

**Published:** 2022-12-22

**Authors:** Chiara Lionello, Claudio Perego, Andrea Gardin, Rafal Klajn, Giovanni M. Pavan

**Affiliations:** † Department of Applied Science and Technology, Politecnico di Torino, Corso Duca degli Abruzzi 24, 10129 Torino, Italy; ‡ Department of Innovative Technologies, University of Applied Sciences and Arts of Southern Switzerland, Polo Universitario Lugano, Campus Est, Via la Santa 1, 6962 Lugano-Viganello, Switzerland; § Department of Organic Chemistry, Weizmann Institute of Science, Rehovot 76100, Israel

**Keywords:** colloidal superlattices, ion dynamics, supramolecular semiconductivity, ionic conductivity, molecular dynamics, coarse-graining, machine learning

## Abstract

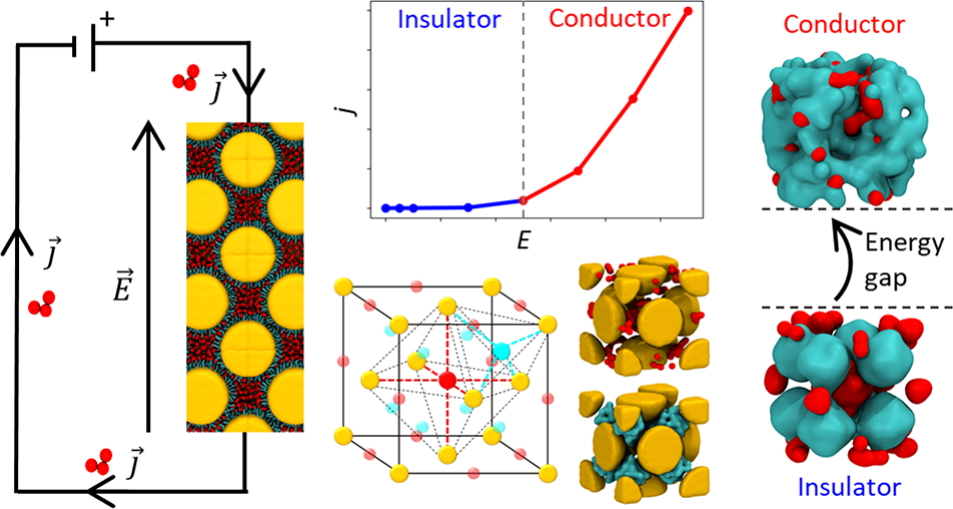

The self-assembly of nanoparticles driven by small molecules or ions may produce colloidal superlattices with features and properties reminiscent of those of metals or semiconductors. However, to what extent the properties of such supramolecular crystals actually resemble those of atomic materials often remains unclear. Here, we present coarse-grained molecular simulations explicitly demonstrating how a behavior evocative of that of semiconductors may emerge in a colloidal superlattice. As a case study, we focus on gold nanoparticles bearing positively charged groups that self-assemble into FCC crystals *via* mediation by citrate counterions. *In silico* ohmic experiments show how the dynamically diverse behavior of the ions in different superlattice domains allows the opening of conductive ionic gates above certain levels of applied electric fields. The observed binary conductive/nonconductive behavior is reminiscent of that of conventional semiconductors, while, at a supramolecular level, crossing the “band gap” requires a sufficient electrostatic stimulus to break the intermolecular interactions and make ions diffuse throughout the superlattice’s cavities.

## Introduction

The self-assembly of ligand-protected nanoparticles (NPs) into colloidal superlattices is attracting great interest.^1−12^ A wide range of supramolecular architectures can be obtained by guiding the self-assembly of the NPs toward various types of crystalline or quasicrystalline aggregates.^1,13−23^ Crystalline NP superstructures have been attained, for example, *via* rational design of NP shape, anisotropy, surface chemistry,^24−29^ or by manipulating the conditions of the self-assembly process.^10,30,31^ Another effective way to control the formation of ordered colloidal superlattices is to use molecular binding units capable of driving NP self-assembly. As an example, the specific recognition between complementary DNA strands grafted onto the NP surface has been used to assemble, with great precision and fidelity, colloidal NP superlattices with various symmetries and fascinating properties.^24,32−38^ Electrostatic interactions have been also widely employed to drive the self-assembly of colloidal particles. For example, the coassembly of oppositely charged NPs or the self-assembly of same-charge colloidal entities using oppositely charged ions (or binders) has allowed the generation of binary crystals,^25,39−41^ homo-NP superlattices,^31,42−44^ or supramolecular fibers, among other materials.^45^ While the NP structure, the NP–NP interactions, self-assembly process, and NP organization in the supercrystals are crucial factors for the properties of these materials,^44,46−48^ in the case of mediated self-assembly, it has been demonstrated how the nature and behavior of the mediating species is also of key importance.^31,45,49−54^

On the one hand, such supramolecular materials are interesting for their fascinating properties (among others, plasmonic, magnetic, and electrical).^55−58^ On the other hand, from a fundamental point of view, NP superlattices are perhaps even more intriguing because they represent, to some extent, larger scale analogues of atomic crystals, where the NPs organize in space as “superatoms.”^59−64^ In general, learning how to master the interparticle interactions in such a way as to obtain colloidal supercrystals with controllable higher scale features is a major goal.^31,40,44,65,66^ However, the atomic/molecular factors underpinning the emergence of collective assembly properties often remain difficult to ascertain.

Pioneering works have shown how the selectivity of DNA interactions can be exploited to assemble NPs functionalized with single-stranded DNA into a variety of superstructures with symmetries typical of a metallic crystal.^38,67−72^ As “atom equivalents,” the NPs in such supercrystals are surrounded by the smaller particles, which keep them together as superelectron equivalents. Experiments and molecular simulations showed how the electron equivalents can break their distribution symmetry around the atom equivalents and move in the superlattice in a way resembling the behavior of electrons in metals.^38,67−73^ Similar intralattice mobility has also been observed in aggregates of gold NPs decorated with a high density of positively charged groups. Cl^–^ counterions coassembled with NPs decorated with a high density of (11-mercaptoundecyl)-*N,N,N*-trimethylammonium (TMA) groups generated colloidal superclusters with semiconductive properties useful for building chemoelectronic circuits.^49,51,54^ More recently, static and dynamic superlattices have been also obtained via mediated assembly of TMA-NPs (atom analogues, AAs) in water using citrate (CIT) or ATP as counterions. Also in this case, rich dynamics of the CIT ions (behaving as electron analogues, EAs) were observed in the TMA-NP superlattices.^31^ The ability of the EAs to move in AA superlattices may suggest intriguing hierarchical analogies with atomic crystalline materials. For example, metal-like transitions^71,73^ can be associated with variations in the EA distribution upon temperature change. Interesting similarities also arise with superionic conductance, in which components of ionic crystals can diffuse across emerging defects in the lattice, which similarly results in significant charge transport.^74−78^ However, it is worth noting that the conductive behavior of metals, semiconductors, or superionic conductors exhibits specific fingerprints, which are nontrivial to ascertain in such higher scale supercrystals.

Here, we show coarse-grained (CG) molecular simulations that allow us to enter in a realistic colloidal lattice model at submolecular resolution and to reconstruct in great detail the mobility of the EAs and key factors that control the conductive properties of the supercrystal. Using FCC superlattices of TMA-NPs (AAs) coassembled with CIT ions (EAs) as a case study,^31^ we designed *in silico* ohmic experiments that enable the systematic study of the ion diffusion throughout the lattice upon the application of an external electric field. This approach provides us with clear evidence of how the ion-driven conductive behavior of such superlattices differs from classical (electron-driven) metallic conductivity by showing features closer to the typical behaviors of semiconductive materials (however, what distinguishes classical semiconductors from NP superlattices is the identity of charge carriers: electrons and CIT ions, respectively). At the high resolution of our CG models (<5 Å), we obtain a clear insight on the collective mechanism underpinning the ionic conduction in our system, which provides further knowledge toward controlling the emerging properties of colloidal supercrystals.

## Results and Discussion

**Figure 1:**
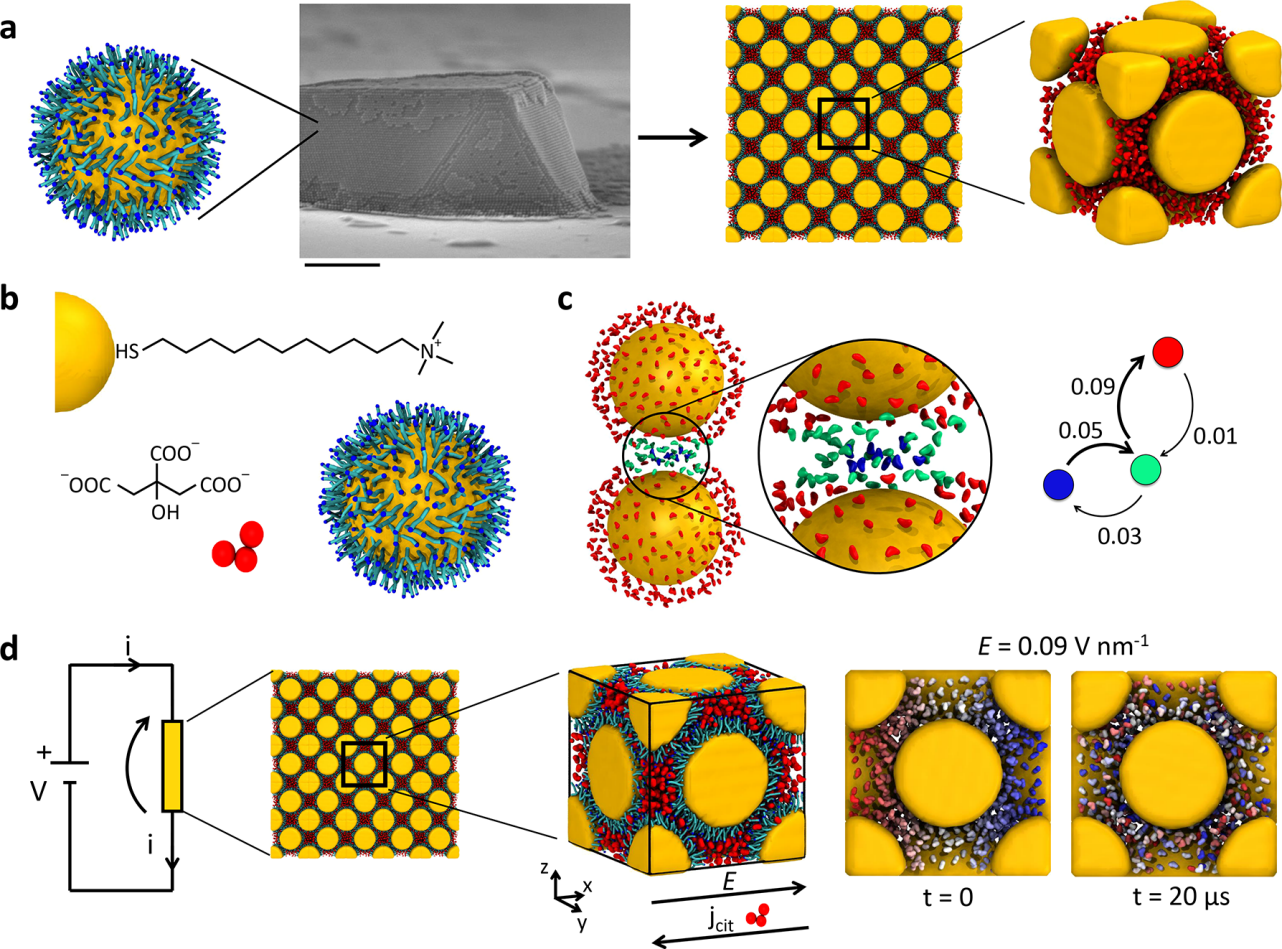
NP–citrate colloidal superlattices and *in silico* ohmic experiments of ionic conductivity. (a) Trivalent citrate ions (CIT) driving the self-assembly of TMA-Au NPs into crystalline FCC superlattices in an aqueous solution (for experimental details see ref (31).) Left: schematic representation of a Au NP. Center: a representative SEM image of a colloidal crystal coassembled from 7.4 nm TMA-Au NPs and citrate trianions. Right: Model of TMA-Au NP superlattice. Au NPs are colored as golden spheres, citrates are colored in red. (b) Structural formulas of the positively charged TMA ligands and of the negatively charged CIT ions, and CG models (resolution <5 Å) of one CIT (red) and one TMA-NP (*d* = 7.4 nm, yellow) decorated with 804 charged TMA ligands (cyan; terminal charges in blue). (c) CG molecular dynamics (MD) simulation snapshot showing CIT ions mediating the interaction between two TMA-NPs (TMA ligands not shown for clarity). Unsupervised clustering of SOAP data extracted from the CG-MD simulation detects three different/distinct CIT environments.^31^ CIT ions at the NP–NP interface are colored blue, CIT ions bound to one NP only are in red. The two CIT domains communicate by exchanging CITs via an intermediate green domain. Inset: detail of the CIT ions close to the NP–NP interface, colored on the basis of their SOAP-detected cluster/state. Right: transition plot showing the dynamic interconnections between the SOAP environments. The numbers on the arrows are normalized transition probabilities (probability that one CIT in a given environment undergoes a transition into another in the time interval used in the analysis).^31^ (d) Left: scheme of the *in silico* ohmic experiments to study the ionic conductivity of the superlattices. Center: CG model of the bulk of an FCC TMA-NP superlattice (unitary FCC cell containing four TMA-NPs, and its replication in space): Au NPs in yellow, TMA ligands in blue, CITs in red. A uniform electric field (*E*) oriented along *x* direction is applied during the MD simulations, and the mobility of the CIT ions is systematically monitored for different *E* intensities. Right: example of motion of CITs during the MD simulation at *E* = 0.09 V nm^–1^. The CIT ions are initially colored on the basis of their *x* position (left, *t* = 0 μs); reshuffling of colors after *t* = 20 μs of MD (right) demonstrates reshuffling and diffusion of the CIT ions.

As a case study of colloidal superlattices composed of large AAs held together by smaller and mobile EAs, here, we focus on the recently reported colloidal supercrystals of positively charged TMA-NPs coassembled with negatively charged, trivalent CIT ions (Figure 1a,b).^31^ At the experimental level, TMA-NPs can be coassembled with CIT ions in water to produce well-defined, compact superlattices, in which the TMA-NPs (AAs) are arranged into an FCC lattice (Figure 1a). All-atom and consistent implicit-solvent CG models (Figure 1b) showed how the large population of −3*e* charged CIT ions allows stabilizing the assembly of two TMA-NPs, overcoming the electrostatic repulsion between the positively charged TMA-NPs.^31^ It is noteworthy that even while stabilizing the NP–NP interaction, the CIT ions were observed to preserve a dynamic behavior (Figure 1c). In such a two-NP model, unsupervised clustering analysis of smooth overlap of atomic positions (SOAP)—high-dimensional descriptors effective in classifying the ionic environments surrounding each CIT molecule along molecular dynamics (MD) simulations on the basis of their level of order/disorder—detected three main CIT environments: CIT ions at the NP–NP interface (Figure 1c, blue), CIT ions interacting with a single NP (red), and an intermediate ionic layer (green). In general, the interface (blue) CIT ions are more static, and the red ones are more dynamic, while the ionic environments are in dynamic communication *via* a continuous exchange of CIT ions in equilibrium conditions. The interconnection plot of Figure 1c (right), which identifies the dynamic CIT transitions between the various environments, shows the allowed transitions in the system.^31^ Such a rich dynamic behavior of the CIT ions fits well with the experiments, thereby allowing the initially formed amorphous aggregates to rearrange into ordered FCC crystalline lattices (within hours).^31^ At the same time, the TMA–NP–CIT superlattice offers an ideal system that can be studied as a supercrystal of AAs surrounded and held together by mobile EAs.

We obtain more direct evidence into what extent such EA mobility in a superlattice produces features typical of, e.g., metallic or covalent crystals by designing an *ad hoc* ohmic *in silico* experiment. We use previously validated implicit solvent CG models, which guarantee relatively high resolution (<5 Å) and good consistency with chemically relevant all-atom models,^31^ by creating a model FCC lattice. The simulated box is composed of four NPs, each decorated with 804 positively charged TMA groups that are arranged in such a way as to produce, when replicated in three dimensions via periodic boundary conditions (PBC), an infinite FCC lattice consistent with the experimentally obtained lattice parameters.^31^ In the FCC cell model, the TMA-NPs are surrounded by 1072 −3*e* charged CIT counterions. In all simulated systems, there are no other ions than CITs. The basic idea is that the application of a homogeneous directional electric field (*E*, oriented along the *x* direction) allows monitoring of the diffusion of CIT ions within the TMA-NP superlattice and reconstruction of the diffusion mechanisms (Figure 1d, left and center). Since PBCs enable the recirculation of CIT ions from one side of the simulation box to the other as they drift along the *E* field, the model mimics a perfect superlattice with an infinite reservoir of CIT ions, providing a suitable setup for the conceptual purpose of the present work. Details on the molecular models and on the simulation protocols are provided in the Methods section. After preliminary minimization and equilibration of the molecular model, we ran a set of MD simulations with *E* = 0 (reference unperturbed case), 0.005, 0.01, 0.03, 0.05, 0.07, 0.09, and 0.11 V nm^–1^. In all these MD simulations, *E* is uniform and oriented along the *x* direction. We explore the behavior of the system at two different temperatures, *T* = 300 K and *T* = 333 K, to study to what extent increasing the thermal agitation increases the mobility of the CIT ions (EAs) and eventually alters the conductive properties of the superlattice. All systems were simulated for 20 μs of MD, during which they were observed to reach a dynamic equilibrium guaranteeing enough data to reconstruct the dynamics and mechanisms of the CIT diffusion.

**Figure 2:**
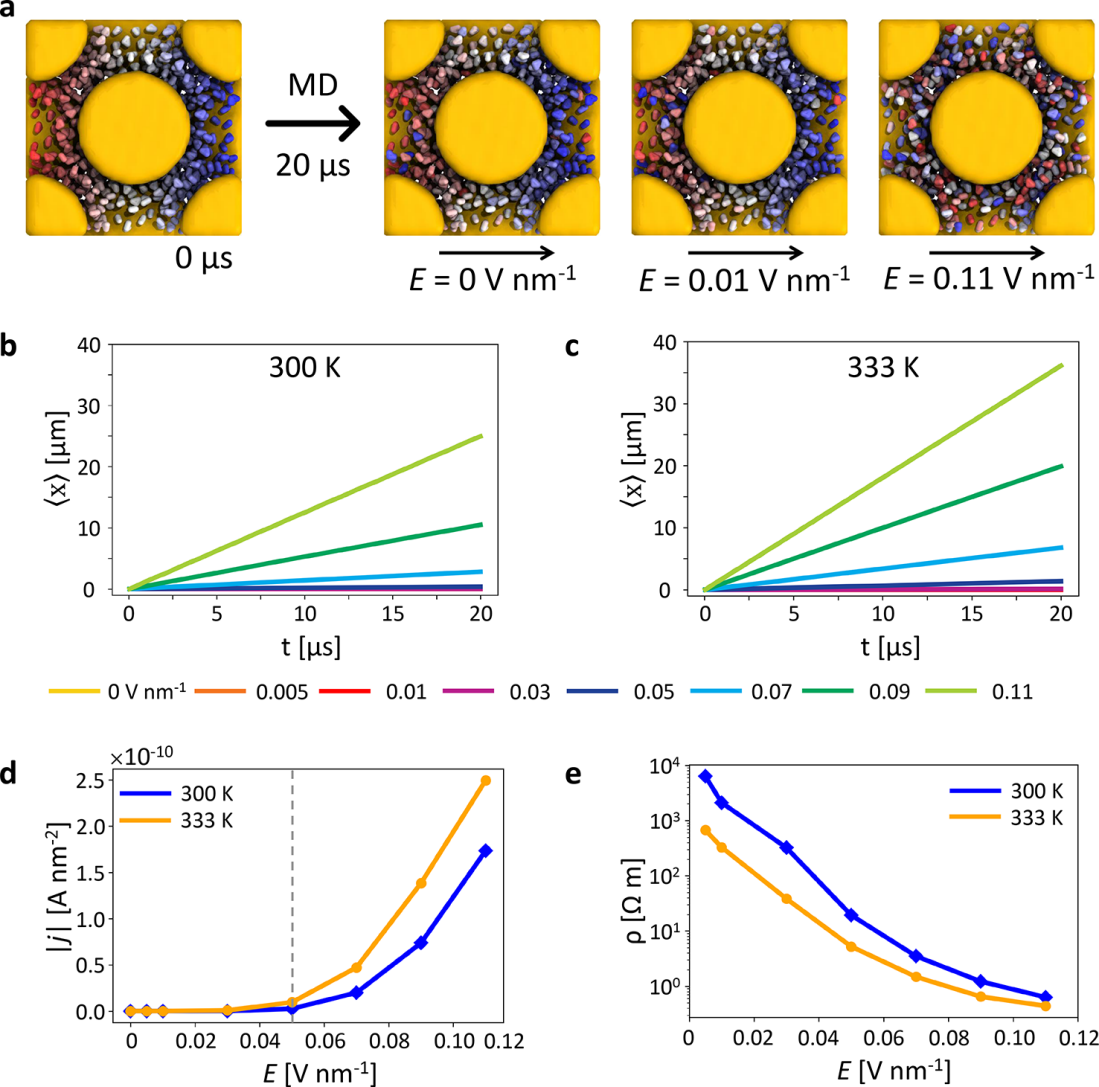
Ionic conductivity of the colloidal crystal. (a) Snapshots at the beginning (left) and after 20 μs of MD simulation (right) at *T* = 300 K for different values of the electrostatic field, *E* (the initial configuration is identical in all simulations). CIT ions are colored according to their initial *x* position at the beginning of the MD (*t* = 0) in the simulation boxes, TMA groups are not shown for clarity. CIT diffusion along the *x* axis is proven by the red–white–blue color reshuffling at the end of the simulations. (b) Average displacement of the CIT ions along the *x* direction (direction of the applied *E*) at *T* = 300 K as a function of MD simulation time. Data are reported for different values of applied *E* (see color legend). (c) Same as (b) at *T* = 333 K. From the slope of the colored lines, it is possible to estimate the average current density *j* in all simulated cases. (d) Current density *j* associated with the CIT diffusion along *x* as a function of *T* and *E*. As the *E* intensity overcomes a threshold value (vertical dashed line), the system switches from nonconductive to conductive regime. (e) Resistivity ρ associated with the CIT current as a function of *T* and *E*.

It is possible, by monitoring the overall CIT ions dynamics at different *E* intensities, to compare the effect of the electrostatic stimulus on the internal dynamics of the EAs. Figure 1d (right) shows how CIT ions, initially colored with a color palette from red to blue, on the basis of their *x* position in the simulation box, reshuffle in the superlattice during the MD at *E* = 0.09 V nm^–1^. The MD snapshots in Figure 2a also demonstrate how at *T* = 300 K the CIT reshuffling changes with the intensity of *E* (all the systems share the same initial configuration): the higher the deviation is from the initial color pattern, the more significant the CIT diffusion is along the *E* direction (*x* axis). In particular, while a weaker *E* (e.g., *E* = 0 and 0.01 V nm^–1^) appears to have little effect on the CIT dynamics, an increase in the *E* intensity (e.g., *E* = 0.11 or 0.09 V nm^–1^) notably enhances the CIT reshuffling during the same CG-MD simulation time. A similar behavior is observed at *T* = 333 K, while in this case the mobility of the CIT ions is, in general, increased with respect to *T* = 300 K because of the stronger thermal agitation (see Supplementary Figure S4).

From the MD simulations, we estimated the average *x* displacement of the CIT ions during the runs (Figure 2b,c ⟨*x* ⟩). The data show how in all cases the systems reach a dynamic equilibrium; that is, the diffusion along the *x* direction reaches a constant rate during the MD (see Methods section for further details). While these data are extracted from an approximated CG model (where, e.g., the *E* is constant in intensity and direction, polarization effects on the solvent molecules or on the Au NPs are not explicitly treated, etc.), and as such should be considered as qualitative, they provide interesting insights, useful for understanding the global behavior of these superlattices. After a short initial transient phase, in all cases where CIT diffusion is activated by the applied *E*, the systems reach a steady state characterized by a constant ionic current. The plots in Figure 2b,c also show how the extent of this ionic drift is proportional to the *E* intensity. From the ⟨*x*⟩ data over MD time, it is possible to estimate the ionic current *j* = *Nqv*_d_, where *N* is the number density of the CIT ions, *q* = −3*e* is the CIT charge, and *v*_d_ is the average drift velocity (in *x* direction) measured in our simulations. Figure 2d shows the absolute value of the current *j* as a function of the intensity of the applied *E*. From the current density *j* measured at the various *E* intensities, it is possible to estimate the resistivity of the superlattice using Ohm’s law, ρ = |*E*/*j*|. The *j* plot shows two different regimes: for *E* lower than ∼0.05–0.07 V nm^–1^ (at *T* = 333–300 K, respectively), the ionic current is relatively negligible and the superlattice is nonconductive, while for higher *E* intensities, the CIT current density increases quasi-linearly with the amplitude of *E*. Accordingly, Figure 2e shows a decrease in the measured ρ for increasing *E* intensities. Together, our results indicate a nonohmic behavior of such superlattices in terms of CIT conduction, which is in sharp contrast with pure metals—typically ohmic conductors. Indeed, these results show a binary nonconductive/conductive behavior as a function of *E*—a behavior reminiscent of that of electronic semiconductors. In these supramolecular systems, the conductivity is ionic and controlled by intermolecular interactions (e.g., CIT–NP and CIT–CIT). In particular, the classical intermolecular interactions between CIT ions and the charged NP surface ligands impose energy barriers that must be overcome at finite temperatures (activation energy) to allow charge (CIT) conduction within these superlattices. We observe that a minimum electric field intensity (*E* = ∼0.05 V nm^–1^) is required to enable ionic hopping between different sites within the crystal (thus allowing conduction). This behavior is, to some extent, reminiscent of that of crossing the band gap in electronic semiconductors, which turns the material from nonconductive to conductive, except that here the charge is carried by relatively large ions, which are restrained by classical intermolecular forces that limit their motion along the electrostatic field (while in electronic semiconductors the electrons are subject to quantum dynamics). We also note that the increase of current density *j* and decrease of resistivity ρ with increasing temperature (Figure 2d,e) underline another difference between such superlattices and pure metals, where increasing temperature typically results in opposite trends (i.e., a decrease in *j* and an increase in ρ). This discrepancy arises from the different physical nature of the “supramolecular” ionic conductivity in our NP superlattices versus electronic conductivity in metals. In the superlattices, thermal agitation provides the CIT ions with sufficient energy to overcome the free-energy barriers determined by intermolecular attraction with the NP ligands, which allows them to cross the “band gap” (as in semiconductors). In metals, the temperature dependence is opposite and is controlled by quantum phenomena, such as phonon scattering. Concerning the conductivity–temperature relationship, we find analogies between our superlattices and superionic conductors,^74−76^ in which temperature increase implies higher disorder and, therefore, higher ion mobility through the sublattice. Similarly, thermal agitation impacts conductivity in our system by releasing the constraints on the ionic carriers, even though the ion dynamics in the studied superlattices differs from that observed in superionic crystals,^76,77^ given the larger scales involved in the phenomenon. It is worth noting that the exact values of the *E* field, density current, or resistivity have little quantitative meaning *per se* in our simulations and should rather be considered qualitatively, since they pertain to an approximated CG model. Yet, the observed trends clearly demonstrate how (i) a binary conductive/nonconductive behavior and (ii) the effect of temperature on conductivity reminiscent of that of semiconductors can be obtained on a larger supramolecular scale in superlattices held together solely by intermolecular interactions. Furthermore, it is interesting to note how the estimated ρ for the TMA-NP superlattice ranges between ∼10^–5^ and ∼10^5^ Ω m, in the typical regime of semiconductor materials. In general, while these superlattices present a behavior more similar to those of semiconductors or superionic conductors than to standard metals, these models also offer the possibility to deeply penetrate into these systems to investigate the mechanisms that control their conductive properties.

The data in Figure 2 provide information on the average conductive behavior of CIT ions in the superlattice in different conditions. Interesting questions arise on the microscopic dynamics of the CIT ions and on the local phenomena/mechanisms controlling the CIT conductivity, including, for example, if the anions are moving in a uniform way, and which CITs diffuse and which ones are more static. We reconstruct the microscopic structural and dynamical behavior of the CIT ions in the superlattice by turning to a data-driven approach, which recently proved efficient in various dynamic supramolecular systems.^31,79,80^ In particular, we center one SOAP vector^81−83^ in the center of mass of each CIT ion in the superlattice model. Within a certain cutoff, the SOAP descriptor provides a high-dimensional analysis of (i) the level of order/disorder in the ionic domains in a TMA-NP FCC supercrystal and (ii) how the CIT ions are organized in space with respect to each other. At each MD simulation, we obtain 1072 SOAP spectra, one for each CIT ion in the superlattice, thereby providing a characteristic fingerprint of the ionic environment surrounding each CIT in the FCC lattice cell. The analysis is repeated for 400 snapshots, taken every d*t* = 50 ns along the equilibrated MD simulation trajectories. We, thus, obtain a SOAP data set composed of a total of 428 800 SOAP spectra. Unsupervised clustering of the SOAP data set, by means of the probabilistic analysis of molecular motifs (PAMM) approach,^84^ allows analyzing and rationalizing the high-dimensional information contained in the SOAP data.

**Figure 3:**
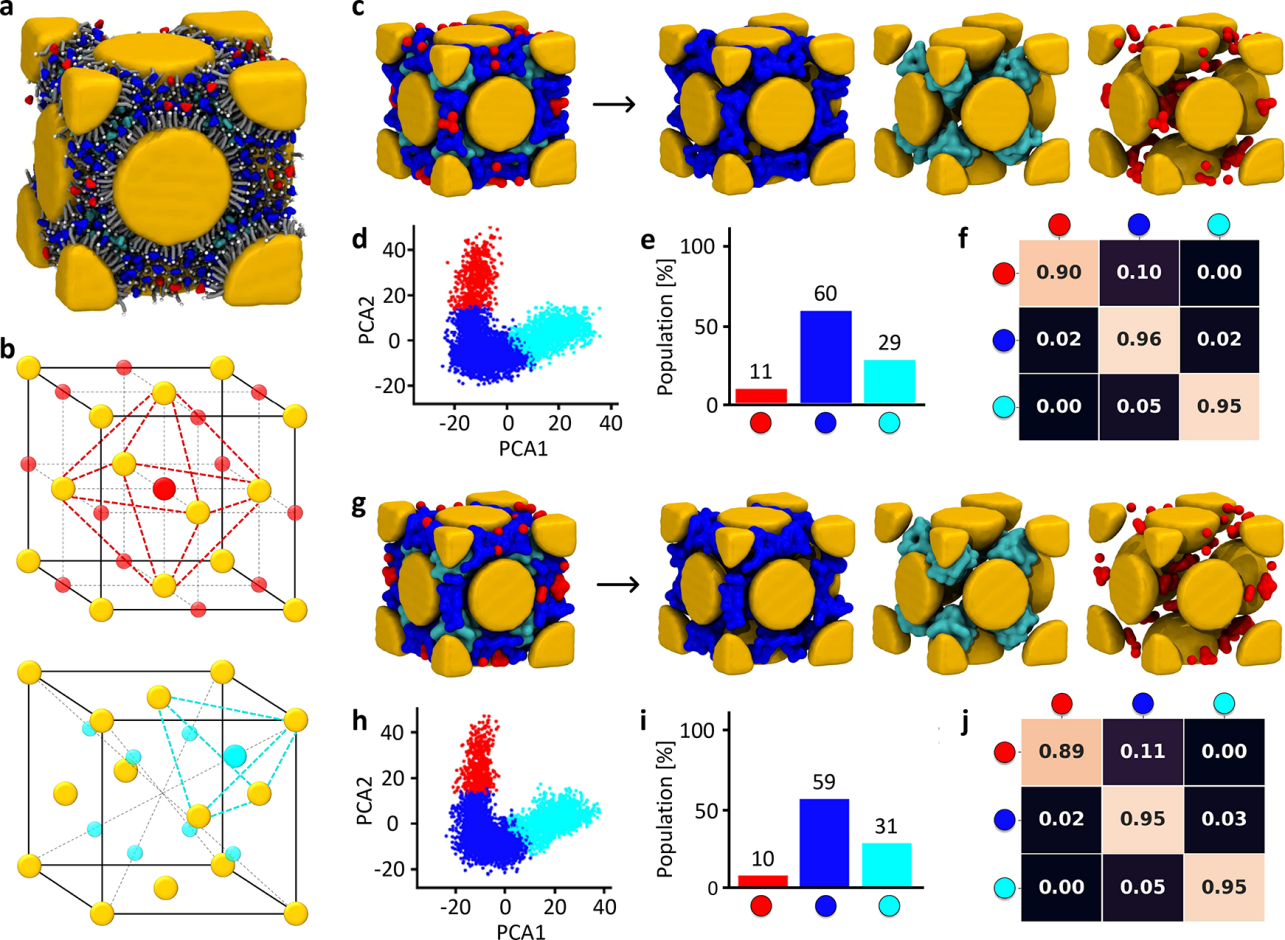
Data-driven detection of dynamic ionic environments in the NP superlattice. (a) Snapshot of the superlattice system at *T* = 300 K and *E* = 0 (after 20 μs of MD simulation). Au NPs are colored yellow and TMA ligands are in gray. CIT ions are colored (blue, red, or cyan) according to their detected SOAP state. (b) Cavities in the FCC superlattice: the octahedral cavities are identified in red (red dots, cavity centers; dashed red lines, cavity sides), and the tetrahedral ones are in cyan. (c–f) SOAP+PAMM analysis identifying the main CIT SOAP environments in the unperturbed systems (*E* = 0) at *T* = 300 K. (c) The SOAP+PAMM analysis detects three main CIT environments at *T* = 300 K: CIT ions at the NP–NP interface are colored in blue, CIT ions in the tetrahedral cavities are in cyan, and CIT ions in the octahedral cavities are in red. (d) Unsupervised clustering (PAMM) of the CIT SOAP data (PCA projection) allows identification of three main SOAP clusters corresponding to the different CIT states/environments in the system. The clustering is performed on the first three principal components (PCs) of the SOAP data set; the projection on the first two PCs is shown. (e) Cluster population histogram. (f) Normalized transition probability matrix indicating the probabilities for a CIT in a given state to remain in that state (*p*_*ii*_, diagonal entries) or to undergo a transition to another SOAP environment (*p*_*ij*_, off-diagonal entries) in the time interval sampled during the analysis (in this case, d*t* = 50 ns). From the off-diagonal transition probabilities, one can also estimate the transition rates as *k*_*ij*_ = *p*_*ij*_/d*t* and a characteristic transition time scale as *t*_*ij*_ = *k*_*ij*_^–1^. (g–j) SOAP+PAMM analysis for the unperturbed systems (*E* = 0) at *T* = 333 K.

PAMM identifies three main clusters in the SOAP data set and identifies different SOAP environments in the FCC superlattice (Figure 3, red, blue, and cyan). CIT ions denoted in a given color belong to the same SOAP cluster, which means that the SOAP ionic environments surrounding them is similar. *Vice versa*, CIT ions identified with different colors belong to different ionic environments. In particular, the Figure 3 panels b, c, and g clearly show how the three detected SOAP environments correspond to well-defined positions within the FCC cell: the CIT ions in between the direct NP–NP interface are colored blue, the CIT in the octahedral cavities in the FCC lattice are in red, and those in the tetrahedral cavities are colored cyan. The visualization of the high-dimensional SOAP data is enabled by employing principal component analysis (PCA) on the entire SOAP data set. Panels d and h of Figure 3 report the projection along the first two principal components (PC) of the SOAP vectors sampled in the *T* = 300 K and *T* = 333 K systems, respectively. The red, cyan, and blue colors indicate the different SOAP-detected motifs, as identified by PAMM clustering. The analysis shows how in the unperturbed superlattice (*E* = 0) the two CIT environments corresponding to the tetrahedral and octahedral cavities are not in direct contact with each other; instead, they are separated by—and both in contact with—the blue environment.

The SOAP analysis is conducted on successive snapshots taken along the equilibrium MD trajectories and keeps track of the individual CIT ions—and the SOAP environments they belong to—at every sampled time step. This analysis allows the reconstruction of the dynamic complexity present in the system at various values of *E* and *T*. We can build the normalized transition probability matrices by monitoring the CIT transitions between the different SOAP environments along the simulation trajectories (see Figure 3f,j). These matrices describe the average probability for a CIT ion belonging to a given SOAP environment to remain there (diagonal entries) or to undergo a transition to another state (off-diagonal entries) in the time interval between two consecutive analyzed snapshots (here, d*t* = 50 ns). While it is worth underlining again the qualitative value of such data extracted from transition probabilities in an approximated CG model, one can easily estimate the average transition rates for a CIT ion in the system to exchange between the various clusters by dividing the off-diagonal probability values by d*t*. For example, at *T* = 300 K, the exchange of one CIT from the octahedral cavities (red) to the NP–NP interface occurs with an average rate (frequency) of ∼0.002 ns^–1^ (characteristic transition time scale of ∼500 ns). From the diagonal entries, we evince that the blue cluster is the most stable one, while the red one is the most dynamic. The blue domain identifies the interface environment between adjacent TMA-NPs in the FCC lattice. There, the CIT ions interact with both NPs and, therefore, “glue” them. Nonetheless, this analysis confirms that such “gluing” action is not static but animated by a certain level of continuous CIT exchange.^31^ All these observations are also valid at *T* = 333 K (Figure 3j), where the internal CIT dynamics in the superlattice appears just slightly more pronounced than at *T* = 300 K. In both cases, the transition matrices demonstrate that at *E* = 0, the CIT ions cannot exchange directly between the octahedral (red) and tetrahedral (cyan) cavities and that these two environments can communicate only indirectly via the more static interface domain (blue).

**Figure 4:**
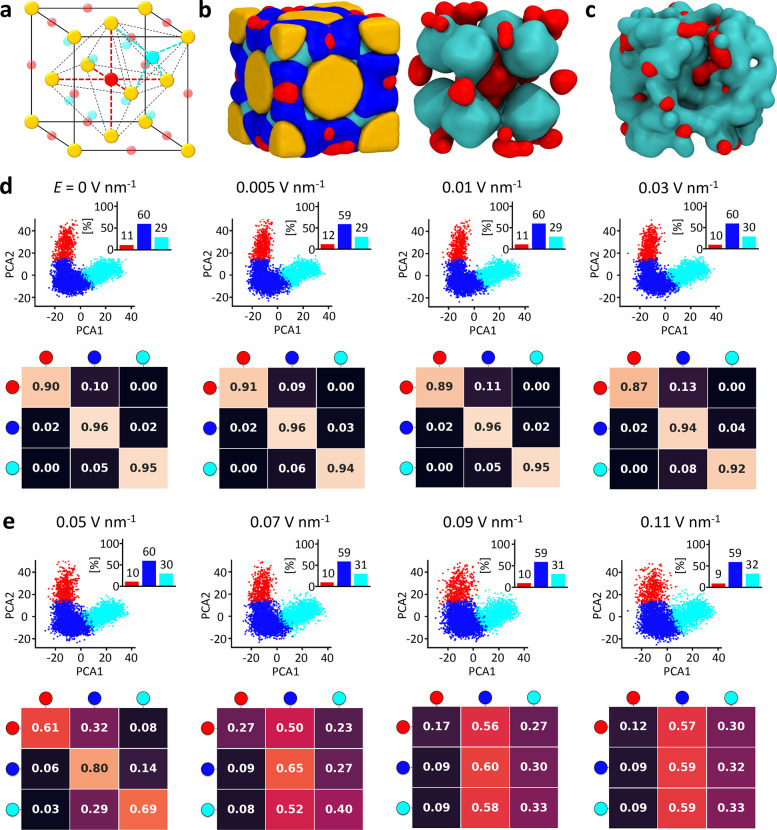
Mechanism of ionic conduction in the NP superlattice at 300 K. (a) The octahedral and tetrahedral cavities in the FCC cell are indicated by red and cyan spheres, respectively. (b) A TMA-NP superlattice: Au NP in yellow, TMA ligands are not shown for clarity, and CIT ions colored on the basis of their SOAP state (left). Right: detail of the CIT ions in the tetrahedral (cyan) and octahedral (red) FCC cavities at *E* = 0. (c) Fusion between cyan and red CIT SOAP environments in the FCC lattice at *E* = 0.11 V nm^–1^, allowing CIT diffusion between the tetrahedral and octahedral cavities of the superlattice (TMA-NPs and blue cluster not shown for clarity). (d,e) Results of the SOAP+PAMM analysis of the superlattice simulation at *T* = 300 K for increasing intensities of *E*. SOAP cluster PC plots and population histograms (top) and transition probability matrices (bottom) for all simulated cases. The analyses show how at *T* = 300 K and for *E* < 0.05 V nm^–1^ all data are similar to the unperturbed case (*E* = 0), while for *E* ≥ 0.05 V nm^–1^, the CIT dynamics change (supramolecular conduction).

It is interesting to compare such data with the same analyses conducted at increasing intensities of *E* (Figure 4). Figure 4b,c shows representative MD snapshots showing the cyan and red CIT ions in the FCC cell in the absence of an electric field (Figure 4b, *E* = 0) versus in the presence of *E* = 0.11 V nm^–1^ (Figure 4c). It can be observed how the red and cyan CIT ions, well-localized in the octahedral and tetrahedral cavities at *E* = 0 (Figure 4a,b), are considerably more reshuffled in the presence of intense *E*. This observation is supported by the quantitative data reported in Figure 4d,e, which clearly distinguishes two distinct regimes: for *E* < 0.05 V nm^–1^, the superlattice preserves very similar features to the nonunperturbed case (*E* = 0), while for *E* ≥ 0.05 V nm^–1^, the CIT dynamics inside the TMA-NP superlattice changes. For *E* < 0.05 V nm^–1^, the transition matrices remain quite similar, thereby indicating that such *E* intensities do not affect the native unperturbed features (in terms of CIT behavior) in the system. In fact, up to this level, the superlattice remains in a nonconductive regime, as seen in Figure 2. It is also noteworthy that the off-diagonal entries connecting the red and cyan SOAP environments in the transition matrices are always 0, which means that in these regimes the CIT ions cannot exchange directly between the octahedral and tetrahedral cavities (Figures 4d, bottom). The situation changes drastically for *E* ≥ 0.05 V nm^–1^. On the one hand, the cluster population histograms do not display significant differences with respect to the simulations at lower *E*, where the interface CITs (blue) constitute ∼60% of all the CIT ions while those in the tetrahedral and octahedral cavities of the lattice constitute ∼30 (cyan) and ∼10% (red) of the CITs, respectively. On the other hand, the transition matrices demonstrate how the diagonal entries drop, and the off-diagonal probabilities increase, which indicates a markedly increased exchange of CIT ions between the SOAP environments. Strikingly, for *E* ≥ 0.07 V nm^–1^at *T* = 300 K, the sole SOAP state preserving a residence probability ≥50% is the blue one, while the red and cyan ones exist as purely transient states. Figure 4e shows nearly identical data for *E* ≥ 0.07 V nm^–1^ at *T* = 300 K. In all cases, the persistence of the blue CIT ions is fundamental for preserving the integrity of the FCC superlattice, while the red and cyan CIT ions allow conduction. *E* = 0.11 V nm^–1^ was found to be a limit for such systems, while higher intensities of *E* were observed to produce instability and destruction of the lattice.

When the lattice enters a conductive regime (Figure 2, *E* ≥ 0.05–0.07 V nm^–1^ at *T* = 300 K), the transition matrices show that the octahedral and tetrahedral cavities in the FCC lattice start to communicate directly (Figure 4e, bottom; red-to-cyan off-diagonal transition probability ≠ 0 and converges to ∼0.30). This finding demonstrates the emergence of ionic gates that, when open, permit the conduction of CIT ions throughout the FCC lattice. As a consequence, when the superlattice enters the conductive regime, the CIT ions at the NP–NP interface act as the “bonding” EAs and keep the lattice together, while the CIT ions in the cavities behave as conduction EAs and diffuse throughout the lattice. To some extent, this nonuniformity in the dynamics of charge carriers is reminiscent of the symmetry breakage and anisotropic distribution of valence electrons and coordination sites around metal atoms in conductive metals, in which some electrons are involved in the stabilization of the material’s structure (atomic bonding), while others (free electrons) allow conductivity in the material. In these colloidal systems, the separation between bound and free CIT charge carriers is less defined than in metals, but nonetheless explicitly evident from our data. Our results demonstrate how, in such NP superlattices, these are collective phenomena that cannot be explained on the basis of, for example, the properties of the CIT ions in the individual SOAP environments in the unperturbed systems. However, this is an emergent behavior that originates from the concerted motions of the ions in response to the stimulus (*E*). This approach provides a demonstration of the importance of studying such complex molecular systems at sufficiently high resolution to track the individual motion of the ions, while at the same time treating sufficiently large scales to observe collective behaviors.^85^

**Figure 5:**
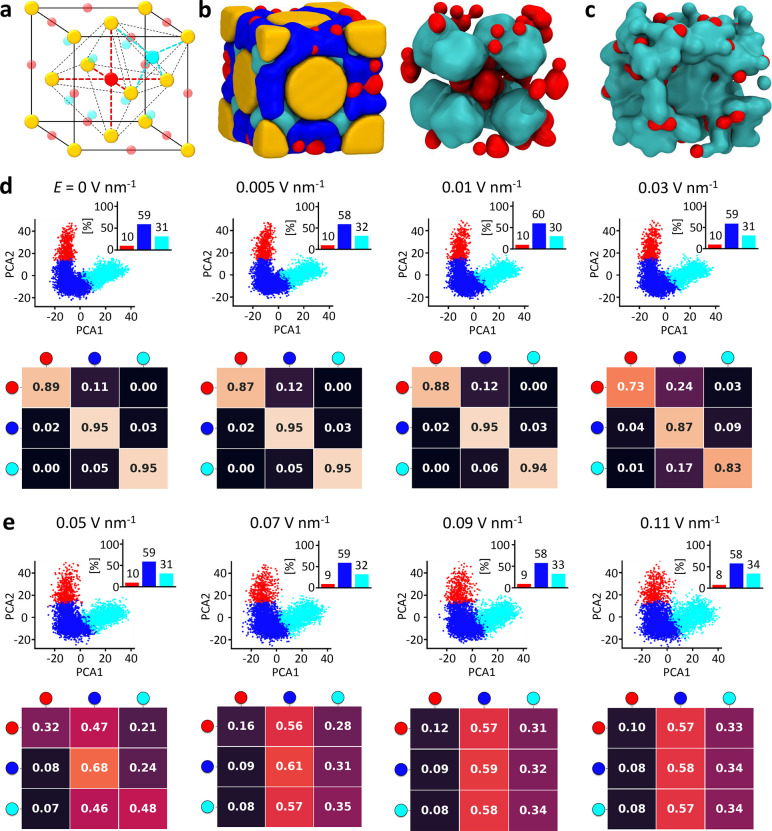
Mechanism of ionic conduction in the NP superlattice at 333 K. (a) The octahedral and tetrahedral cavities in the FCC cell (red and cyan spheres). (b) Left: TMA-NP superlattice (Au NP in yellow, TMA ligands not shown for clarity, CIT ions colored on the basis of their SOAP state). Right: CIT ions in the tetrahedral (cyan) and octahedral (red) FCC cavities at *E* = 0 and *T* = 333 K. (c) Fusion between cyan and red CIT SOAP environments in the FCC lattice at *E* = 0.11 V nm^–1^, allowing CIT diffusion between the tetrahedral and the octahedral cavities of the superlattice (TMA-NPs and blue cluster not shown for clarity). (d,e) Results of the SOAP+PAMM analysis of the superlattice simulation at *T* = 333 K for increasing intensities of *E*, with SOAP cluster PC plots and population histograms (top) and transition probability matrices (bottom) for all simulated cases. The analyses show how at *T* = 333 K for *E* < 0.03 V nm^–1^ all data are similar to the unperturbed case (*E* = 0), while for *E* ≥ 0.03 V nm^–1^ the CIT dynamics change (supramolecular conduction).

Figure 5 shows the results of the SOAP+PAMM analysis for the same systems at *T* = 333 K. We note an overall similar behavior to that observed at *T* = 300 K, with slight differences, coherent with the conductivity results of Figure 2 related to increased thermal agitation. The qualitative insight provided by simulation snapshots (Figure 5b,c) indicates that the localization of the citrates for the two limiting cases of field amplitude (*E* = 0 and *E* = 0.11) is substantially the same as in the *T* = 300 K case. Also at this temperature, the population of the three molecular motifs depends very weakly on the applied field, with the blue state being dominant. The “blue” CIT ions persist even in the conductive regime (diagonal entry converging to ∼0.58), which is fundamental to preserve the FCC lattice integrity. Also in this case, we observe the same features in the characteristic supramolecular ionic semiconductivity of the superlattice, with the conductive regime originating from the emergence of direct ionic gates allowing the diffusion and transport of CIT ions (EAs) throughout the tetrahedral and octahedral cavities present in the TMA-NP (AAs) lattice. As a unique difference, in this case, we note that activation of the CIT conduction requires slightly weaker values of applied electric field (*E* = 0.03 V nm^–1^) than at *T* = 300 K, which is in line with the data of Figure 2d,e. This result suggests that, in this case, a lower electric stimulus is required to cross the supramolecular ”band gap” because the breakage of the CIT–NPs interactions is facilitated by the increased thermal agitation.

## Conclusion

Identifying analogies between the rules/laws that govern the properties of materials across different length scales (e.g., from the atomic- to the nano- and macro-scale) is perhaps one of the fundamental aims of nanoscience and represents a prime objective toward the design of controllable self-assembled materials. On the one hand, materials such as colloidal superlattices present similarities to atomic crystals. On the other hand, the differences in fundamental driving forces that characterize these systems and the difficulty of unambiguous objective investigations make it difficult to understand to what extent the properties of supramolecular materials may really resemble those of atomic and molecular ones.

Stimulated by recent reports on the quasi-metallicity of colloidal superlattices,^71−73^ we have designed an *ad hoc* computational approach to investigate the supramolecular conductive behavior of colloidal crystals coassembled from TMA-functionalized Au NPs and CIT ions (Figure 1)^31^ in which the current is carried by the CIT ions. We, thus, aim at investigating whether—and to what extent—the mobility of ionic species and the superlattice’s responsiveness can mimic the conductivity behavior typical of metals, semiconductors, or insulators. We investigate these specific colloidal crystals as a representative case of larger NPs that arrange into an ordered FCC lattice (AAs) surrounded by a large number of smaller counterions (EAs) that can move in the supercrystal. We use a CG molecular model of FCC lattice that, while approximated, allows studying the lattice in its complexity and retains the essential physical features of the system, thereby providing us with fundamental indications on the behavior of such materials (Figure 1b–d). Through the study of the behavior of the superlattice under the influence of an external electrostatic field, we obtain data explicitly demonstrating how these TMA-NP crystals possess a supramolecular semiconductive character, which allows conduction of CIT ions as charge carriers above a threshold *E* intensity of activation (Figure 2). In this case, the current is generated by the motion of the CIT ions that, unlike free electrons in metals, is triggered by and follows classical motion and statistics.^67,71^ Our *in silico* ohmic experiments show how, for weak values of the external field *E*, no current is generated, but when the *E* intensity overcomes a certain threshold value, an ionic density current proportional to the external field is generated in the system (Figure 2). We observe a transition from a nonconductive to a conductive regime reminiscent of that of semiconductors, while in such supramolecular systems crossing the “band gap” requires a sufficient *E* intensity to break the electrostatic interaction of the CIT ions with the TMA-NP surface and having free ions that diffuse in the system (Figure 2d).

Unsupervised machine learning of the ionic environments that emerge within the superlattice^31,79,80^ allowed the detection of three distinct ionic domains that differ in terms of structural order, persistence, and dynamics (Figure 3). In particular, the CIT ions at the NP–NP interface work as bonding EAs to stabilize the FCC lattice. Conversely, the CIT ions in the octahedral and tetrahedral cavities of the FCC lattice are more dynamic and prone to behave as charge carriers (Figures 4 and 5). Reconstruction of the microscopic dynamics of CIT ions in the system correlates the transition to the conductive regime with the emergence of ionic gates connecting the tetrahedral and octahedral cavities in the FCC superlattice through which the CIT charge carriers can diffuse. The transition matrices of Figures 4 and 5 demonstrate a supramolecular behavior reminiscent of the mechanism of conduction for metals. In particular, in the conductive regime, the “blue” CIT ions at the NP–NP interface interconnect the NPs and keep the lattice stable, while the CIT ions in the cavities behave as the conductive EAs.

Despite the approximations in our CG molecular model (necessary to simulate the relevant space- and time-scales for an exhaustive characterization of the collective mechanisms underpinning the supramolecular conductive process), even from a purely qualitative standpoint our simulations clearly demonstrate how the classical interactions involved in colloidal supercrystals can give rise to complex emergent charge transport behaviors with nonlinear dependence of the material resistivity on the variable external stimulus. In particular, the nonohmic behavior demonstrated in Figures 2, 4, and 5 indicates that the nonconductive/conductive transition is related to the fact that an activation free-energy barrier must be overcome for the conduction process, that is, the electrostatic NP–CIT interactions must be broken to release CIT ions for conduction. The presented approach can be applied to study and rationalize the ionic conductive properties in different kinds of colloidal supercrystals. While we do not aim to provide quantitative comparisons with actual semiconductive materials or devices, it is interesting to note that the trends seen in our results suggest that binary nonconductive/conductive properties can, in principle, also emerge in supramolecular systems ruled by noncovalent interactions. Such evidences hint at possible future applications exploiting these features, similar to how electronic semiconductivity gave rise to logic devices. These results also greatly improve our understanding of how, in such complex molecular systems, concerted dynamics and stimuli-responsive ensemble-properties may emerge from collective molecular behaviors in a way that can be understood only by studying the effect of the stimulus on large ensembles of discrete individual interacting entities.

## Methods

### Coarse-Grained Molecular Dynamics Simulations Protocol

The CG models for the TMA-Au NPs and for the citrate ions studied herein were reported in our previous work.^31^ As described in detail in ref (31), these models were developed on the basis of the results of all-atom simulations of interacting TMA-Au NP subsections in citrate-rich water solvent. Explicit and implicit solvent representations of the system were proposed on the basis of the MARTINI force field,^86,87^ where a CG bead accounts for 3–4 heavy atoms (resolution of ∼5 Å). In particular, we considered the implicit solvent model, which is based on the “dry” MARTINI force-field.^87^ This level of resolution is sufficient to explore the submolecular dynamics of complex supramolecular aggregates while being able to reach the relevant sizes and time scales that characterize their collective, macroscopic behavior (see, e.g., refs (31, 88, and 89)). It is worth noting that the information extracted from such approximated models should be considered qualitative. However, the results obtained from the proposed simulations can be still reliably used to compare the dynamics of the system subjected to different external conditions and to draw indicative trends of the resulting dynamic behavior, as shown in our previous work^31^ and in the present article.

Every studied system comprises four TMA-Au NPs and 1072 CIT^3–^ molecules. Each NP has a gold core (∼7.4 diameter; 12 527 atoms interacting via Lennard-Jones interactions and arranged in FCC lattices; see ref (90) for details) and a ligand shell [804 positively (+1) charged TMA ligands bound to the NP surface via harmonic potentials of length 0.395 nm and force constant *k* = 1.0 × 10^4^ kJ mol^–1^ nm^–2^]. The TMA ligands are modeled using the Martini CG scheme^86^ that models the ligands as 13 heavy atoms using five CG beads and interacting with properly parametrized harmonic bonds and LJ potentials.^31^ CIT ions are modeled as three bound CG beads, with each carrying one negative charge (the total charge of each CIT ion is −3*e*). Complete parameters of the models used in these simulations are available at https://zenodo.org/record/7437648. The number of citrates in the system was set to neutralize the total charge of TMA-Au NPs. We created the conventional superlattice unit cell by building on the simulations from our previous work. In particular, we determined the distance at which the NPs self-assembled by measuring the spacing between the two NPs’ centers of mass (see Figure S1). Since we know that the NPs self-assemble into an FCC lattice [from the small-angle X-ray scattering (SAXS) data^31^], we arranged the four TMA-Au NPs accordingly. The resulting CG-MD simulation cell has an initial size of *L*_*x*_ = *L*_*y*_ = *L*_*z*_ = 14.35 nm. We modeled an infinite superlattice by applying periodic boundary conditions (PBC) in all directions. Given the size of the cell, some residual finite size effects could not be completely excluded.

All CG molecular dynamics (CG-MD) simulations were conducted with GROMACS^91^ software (version 2018.6) by integrating the dynamics of the CG particles via Langevin equations of motion. At first, we ran 20 ns of NVT simulation (constant number of particles, volume, and temperature) to thermalize the system at either *T* = 300 K or *T* = 333 K. During this equilibration phase, we used a stochastic temperature coupling time of τ_*T*_ = 2 ps. Then, we conducted 100 ns of NPT simulation (constant number of particles, pressure, and temperature) to allow the crystal equilibration at a constant pressure of *p* = 2 × 10^–6^ bar via the Berendsen barostat method^92^ with τ_p_ = 10 ps and temperature coupling time of τ_*T*_ = 2 ps. Finally, we employed the same NPT scheme to perform 20 μs long production runs at *T* = 300 K or *T* = 333 K and *p* = 2 × 10^–6^ bar. We used a 20 fs integration time step, which is standard in Martini models.^86^ In the CG-MD simulations with the external field, a constant, uniform electrostatic field along the *x* direction was applied with different values of the amplitude *E* ranging from 0.005 to 0.11 V nm^–1^. We did not observe any instability of the FCC lattice up to *E* = 0.11 V nm^–1^; however, given the limited size of the system, we cannot exclude that at high *E* values other crystal structures are more favorable than the FCC structure. Sampling of this kind of out-of-equilibrium crystal rearrangement is prohibitive with our model. Nonetheless, the assumption that the crystal remains in the FCC structure, even under intense electrostatic fields, should not interfere with the general purpose of our analyses—that is, the observation of a supramolecular semiconductive-like behavior.

### Study of Citrates’ Dynamics

We performed multiple analyses in order to characterize in detail the structure and dynamics of the citrates inside the lattice model. First, we calculated the average *x* displacement of the anions during the simulation time. Specifically, at every time step we averaged the positions of all CITs in each of the simulated systems (different combinations of *T* and *E*). PBC discontinuities in the ion routes were removed to assess the total movement. From the average displacement of the CITs, we calculated the ionic current density, *j* = *Nqv*_d_, where *N* is the number density of the CITs, *q* = −3*e* is their charge, and *v*_d_ is the average drift velocity. From *j*, we could calculate the resistivity of the material, ρ = |*E*/*j*|.

In order to better characterize the dynamics of the CITs by unveiling their intrinsic diversity, we applied a data-driven approach to identify the different molecular states populated by the anionic species,^79,80^ akin to the analysis reported in our previous study on interacting TMA-Au NPs.^31^ The approach is based on the usage of smooth overlap of atomic positions (SOAP)^81^—high-dimensional descriptors that classify the molecular arrangement of each molecule by encoding its atomic/molecular environment. In all our analyses, we computed the SOAP descriptors associated with each CIT ion. We, thus, had a single SOAP species, and the detection of the molecular motifs in the system depended solely on the relative spatial displacement of the CIT ions within the chosen SOAP cutoff (rcut). In particular, at each sampled MD step, we computed the SOAP spectra of each center of mass for all CIT ions (which is a fingerprint descriptor of the level of order/disorder in the displacement of the surrounding CITs centers of mass) within a certain cutoff. We performed this SOAP analysis using the Python package DScribe^93^ using rcut = 65 Å (rcut = 65 Å was found to be the best compromise between computational cost and the level of detail/information; see Supporting Figure S8) and nmax,lmax = 8.

For all the collected CG-MD trajectories, we computed the SOAP vectors of every CIT ion with a sampling stride of d*t* = 50 ns, thereby generating a comprehensive data set of SOAP information associated with the CITs at different temperature (*T* = 300 and 333 K) and electrostatic drive (*E* ranging from 0.005 to 0.11 V nm^–1^) conditions. We then rationalized this high-dimensional data set by applying a clustering algorithm that identifies the most probable molecular states of the anions, thereby allowing the characterization of their structure and dynamics. The dimensionality of the database was first reduced by means of principal component analysis (PCA), which retains only the first three principal components. In this way, we obtained a less demanding data treatment with a contained loss of accuracy.^79^ PCA was performed using the Python package Scikit-Learn.^94^ We then processed the resulting data set of three-dimensional descriptors by means of a density-base unsupervised clustering scheme named probabilistic analysis of molecular motif (PAMM).^84^ This step allowed classification of all the sampled configurations of the CITS within the crystal according to a set of molecular motifs, that is, the most probable structural arrangements. The identification and definition of such molecular motifs is based completely on the data set of collected system conformations. CIT anions were classified into three different states: the CITs at the interface between the two NPs (blue in all figures), those located in the octahedral cavities (red), and those in the tetrahedral cavities (cyan). It was possible, by using this molecular motif classification, to quantify the percentage of population of the three different states, as well as the probability of transition between them (along the collected CG-MD trajectories), thus obtaining information on the structure and dynamics of the CITs under different temperature and electrostatic drive conditions.

## Data Availability

Complete data and materials pertaining to the molecular simulations conducted herein (input files, model files, raw data, analysis tools, etc.) are available at: https://zenodo.org/record/7437648. Other information needed is available from the corresponding author upon reasonable request.

## Supporting Information Available

Additional details regarding the realization of the simulated system, the motion of the system, and other results related to the unsupervised analysis (PDF)
