# Supplementary material for: Supramolecular Semiconductivity through Emerging Ionic Gates in Ion–Nanoparticle Superlattices
Source: ACS Nano. 2022 Dec 22;17(1):275–87. doi: 10.1021/acsnano.2c07558 (PMC9835987; doi:10.1021/acsnano.2c07558)
Supplement: Supplementary file 1 — nn2c07558_si_001.pdf [file nn2c07558_si_001.pdf]

Supporting Information for: Supramolecular Semiconductivity through Emerging Ionic Gates in Ion-Nanoparticle Superlattices

Chiara Lionello[1], Claudio Perego[2], Andrea Gardin[1], Rafal Klajn[3], and Giovanni M. Pavan[1,2,*]

[1]Department of Applied Science and Technology, Politecnico di Torino, Corso Duca degli Abruzzi 24, 10129 Torino, Italy
[2]Department of Innovative Technologies, University of Applied Sciences and Arts of Southern Switzerland, Polo Universitario Lugano, Campus Est, Via la Santa 1, 6962 Lugano-Viganello, Switzerland
[3]Department of Organic Chemistry, Weizmann Institute of Science, Rehovot 76100, Israel
[*]corresponding author: Giovanni M. Pavan (giovanni.pavan@polito.it)

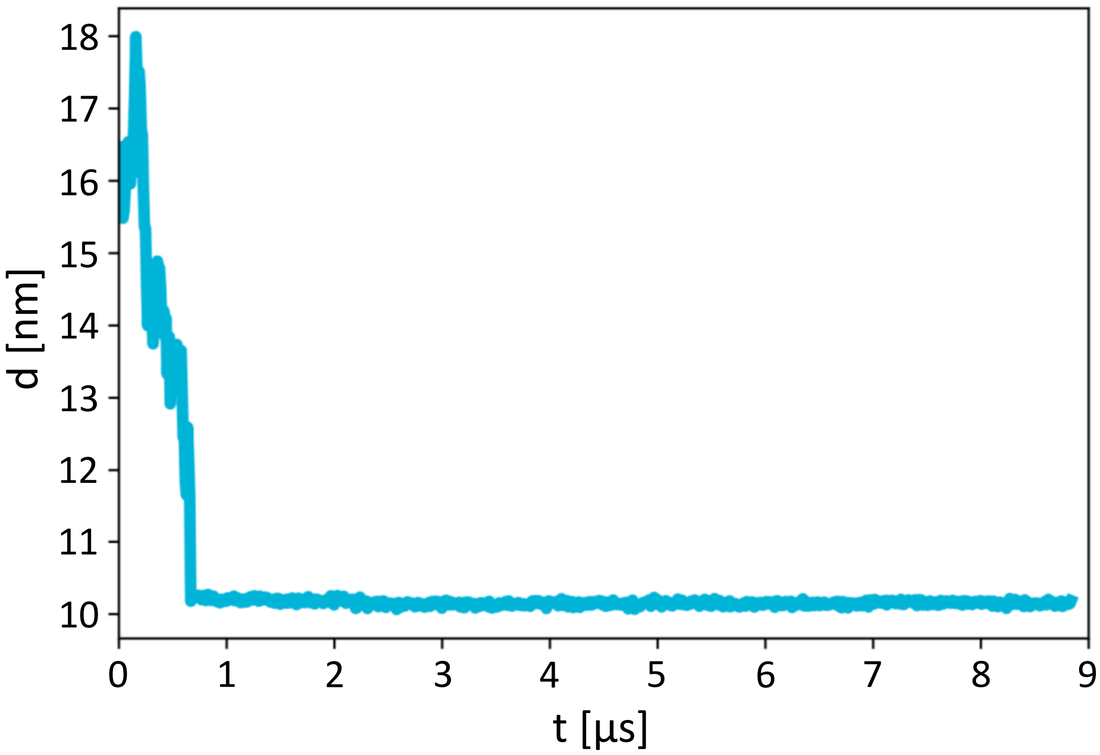

Figure S1. Distance between the center of mass of two self-assembled nanoparticles over time. The mean distance is 10.15 nm.

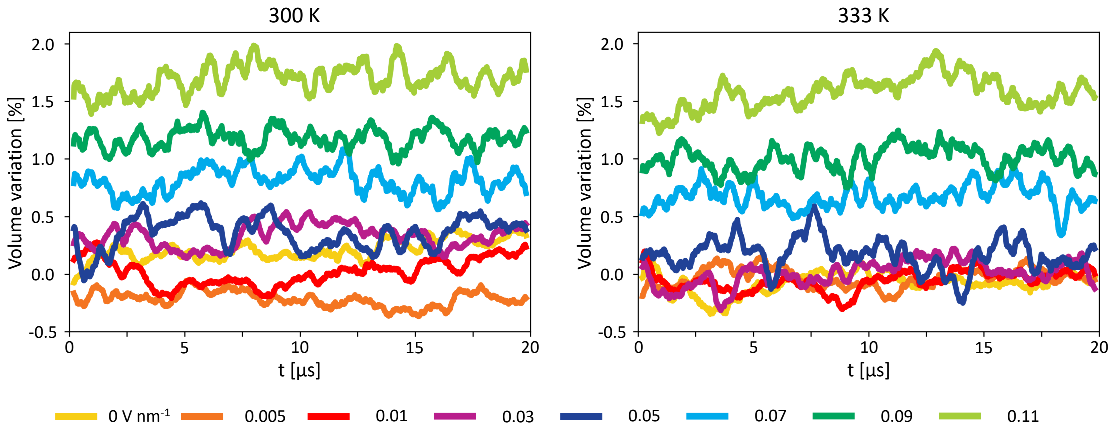

Figure S2. Percentage variation of simulation box volume over time for all the systems studied. Since the volume is not varying, the simulation can be considered at the equilibrium state.

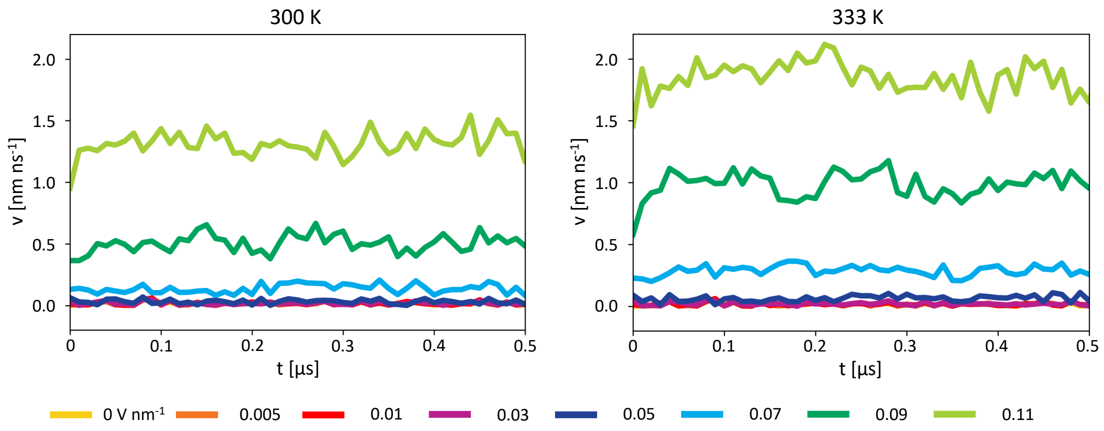

Figure S3. A zoom at the first 500 ns of simulations. In every system the velocity increases during the initial nanoseconds and, immediately thereafter, reaches a plateau (equilibrium).

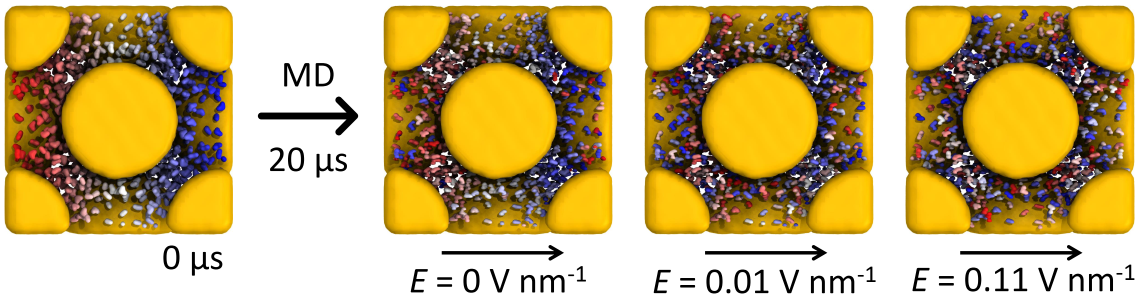

Figure S4. Snapshots at the beginning (left) and after 20 μs of MD simulation (right) at $T = 333$ K for different values of the electrostatic field, E (the initial configuration is identical in all simulations). CIT ions are colored according to their initial x position at the beginning of the MD ($t = 0$) in the simulation boxes; TMA groups are not shown for clarity. CIT diffusion along the x axis is proven by the the red-white-blue color reshuffling at the end of the simulations.

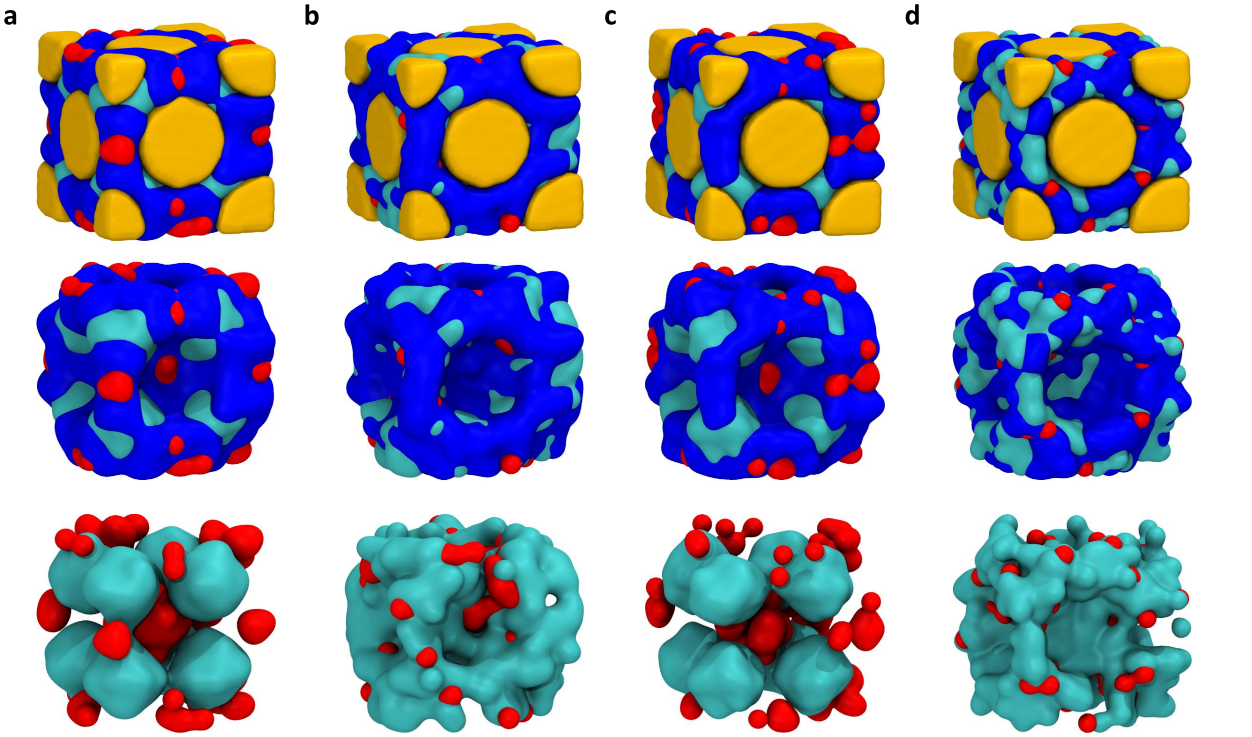

Figure S5. Molecular motifs identified for a representative configuration of the system at the following temperatures and electrostatic fields: a) $T = 300$ K and $E = 0\ Vnm^{-1}$, b) $T = 300$ K and $E = 0.11\ Vnm^{-1}$, c) $T = 333$ K and $E = 0\ Vnm^{-1}$, and d) $T = 333$ K and $E = 0.11\ Vnm^{-1}$. Citrates are colored according to their molecular state. Top: representation of the entire system; centre: the nanoparticles are removed for clarity; bottom: only red and cyan clusters are showed.

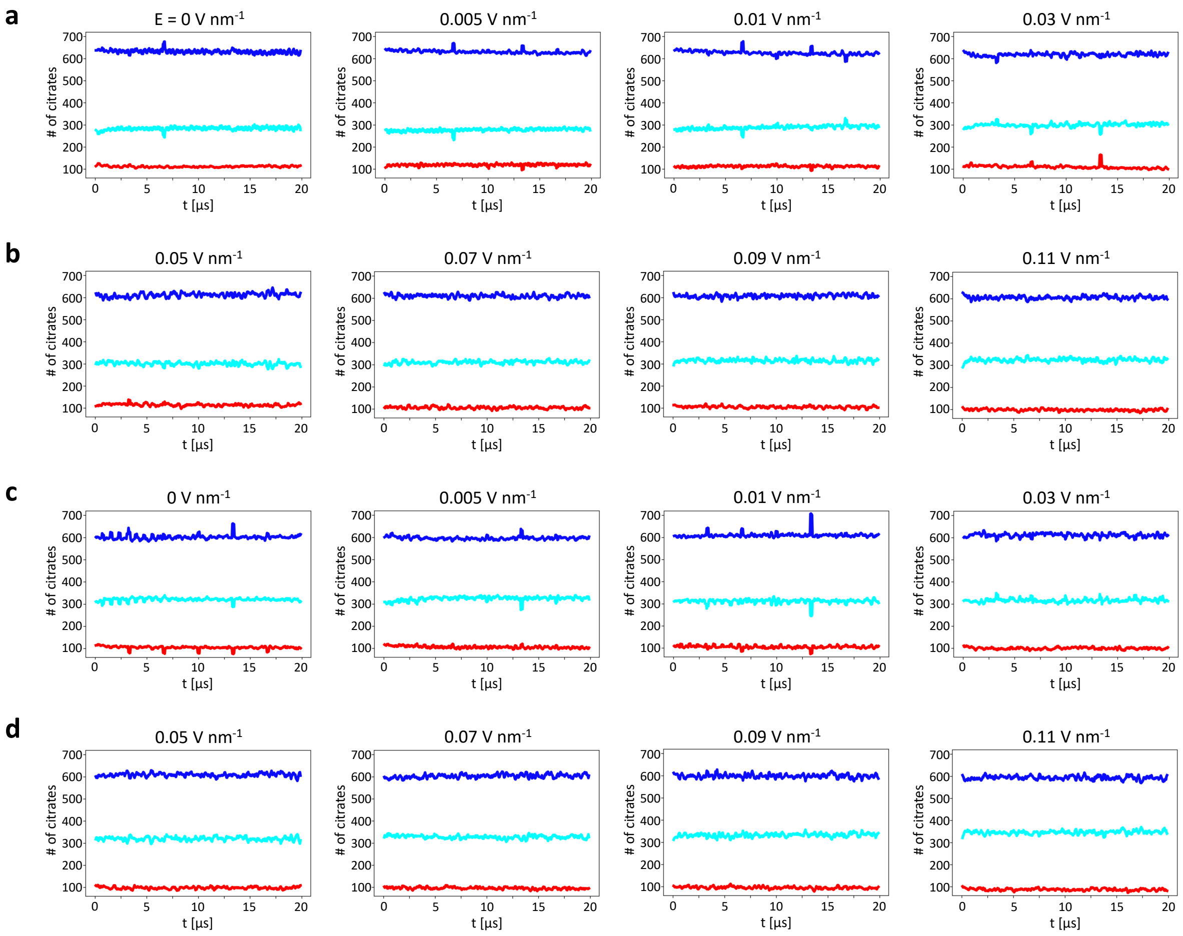

Figure S6. Number of citrates in every cluster over time. a) and b) refer to temperature T = 300 K, while c) and d) refer to T = 333 K.

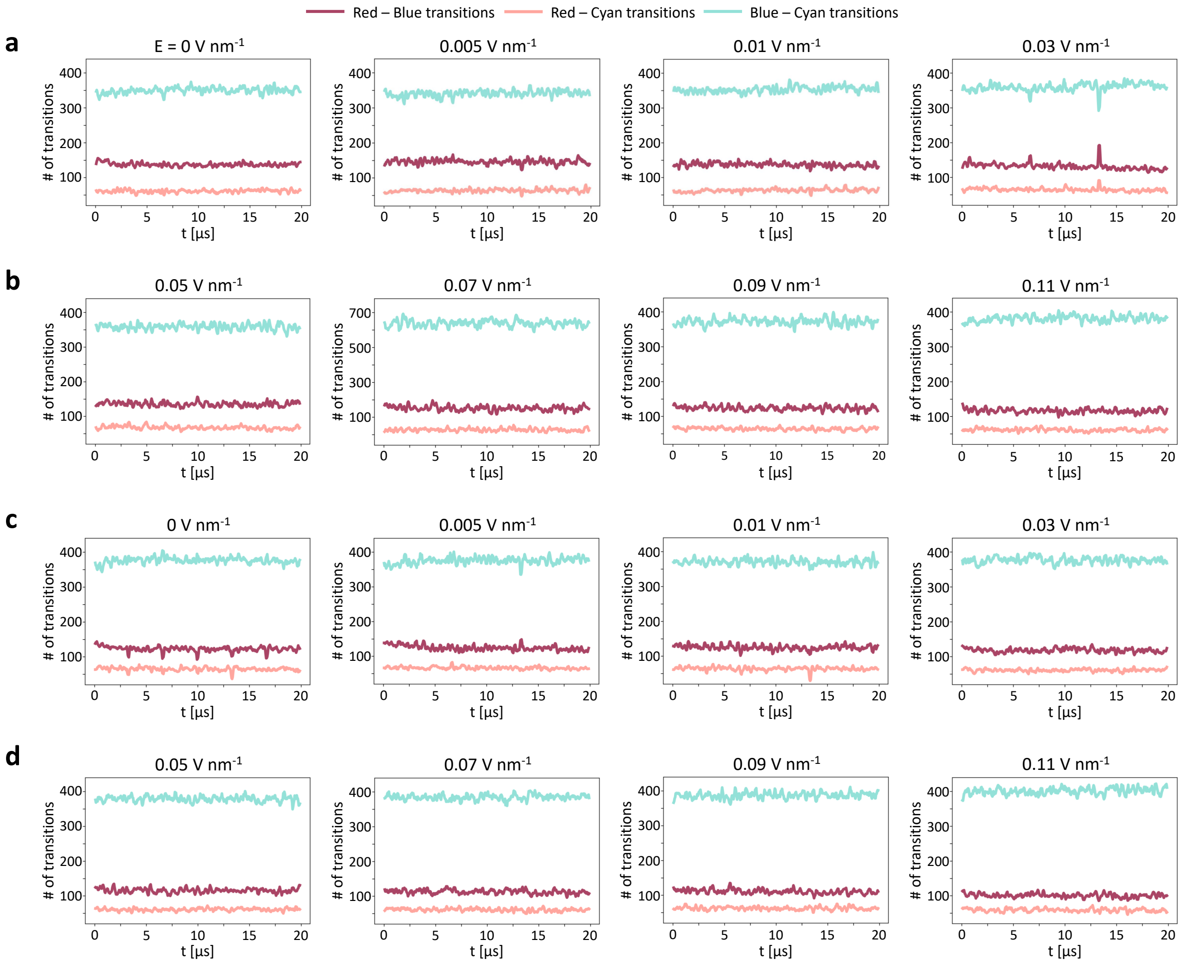

Figure S7. Number of transitions between CIT environment in time. a) and b) refer to temperature $T = 300\ K$, while c) and d) refer to $T = 333$ K.

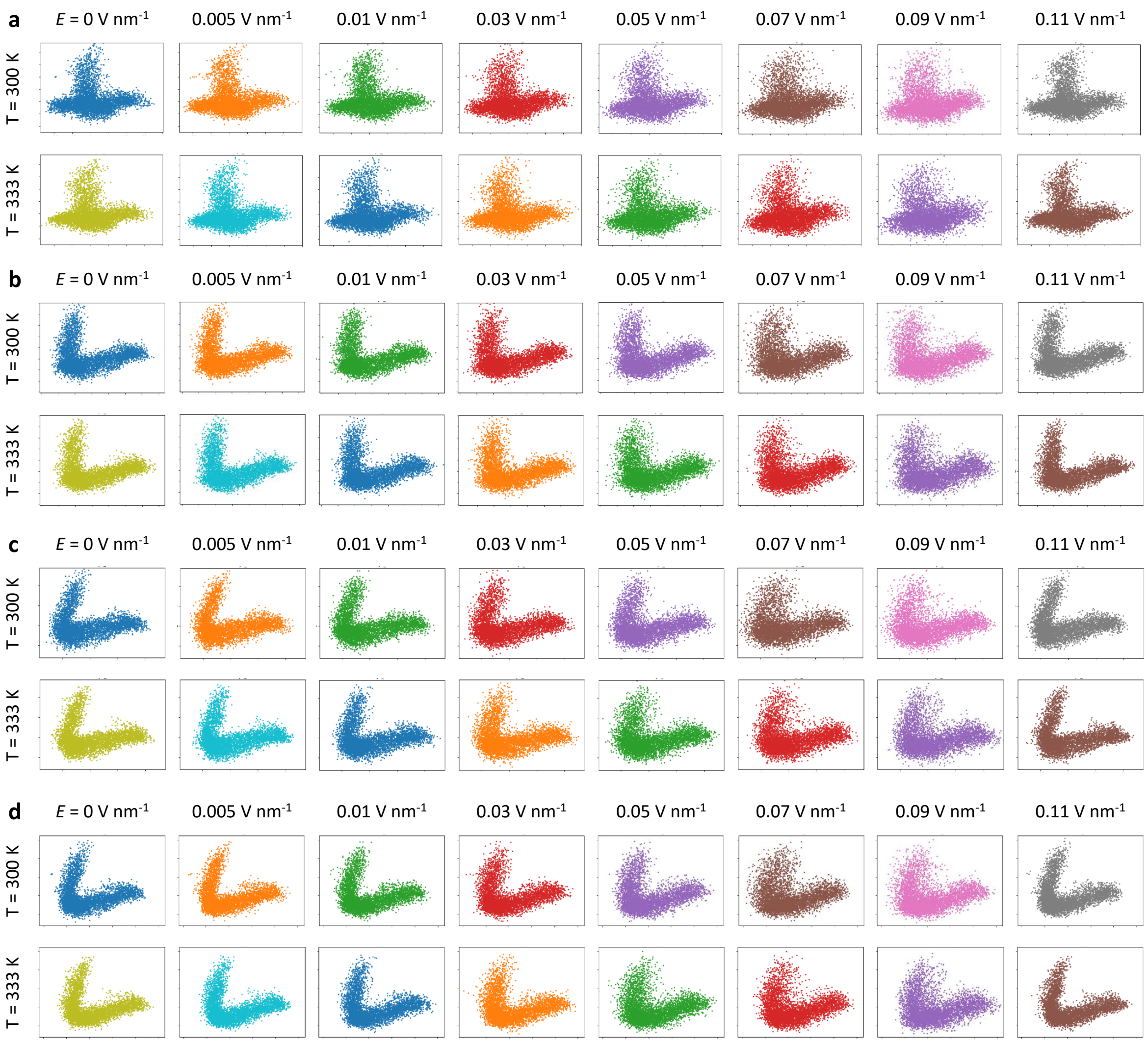

Figure S8. Projection of the SOAP spectra along the first two principal components (PC1 and PC2) for the various systems studied in this work ($T = 300$ and 333 K, variable E). The cutoff radius for the SOAP analysis (rcut) is varied in the different panels:**a** rcut=5.7 nm, **b** rcut=6.5 nm, **c** rcut=7.3 nm, **d** rcut=8.0 nm. The shape of the PCA projections is stable for rcut 6.5 nm, meaning that rcut=6.5 nm is the best compromise between computational cost (which increases with rcut) and information retained by the analysis.
